# 513. Cefiderocol Retains *in vitro* Activity Against Enterobacterales Non-Susceptible to β-lactam-β-lactamase Inhibitor Combinations

**DOI:** 10.1093/ofid/ofae631.165

**Published:** 2025-01-29

**Authors:** Boudewijn L DeJonge, Sean T Nguyen, Jason J Bryowsky, Joshua Maher, Rodrigo E Mendes, Christopher M Longshaw, Miki Takemura, Yoshinori Yamano

**Affiliations:** Shionogi Inc., Florham Park, New Jersey; Shionogi Inc., Florham Park, New Jersey; Shionogi Inc., Florham Park, New Jersey; JMI Laboratories, North Liberty, Iowa; Element, Iowa City (JMI Laboratories), North Liberty, IA; Shionogi B.V., London, England, United Kingdom; Shionogi & Co., Ltd, Toyonaka, Osaka, Japan; Shionogi & Co., Ltd., Toyonaka, Osaka, Japan

## Abstract

**Background:**

Over the past ten years β-lactam-β-lactamase inhibitor combinations, such as ceftazidime-avibactam (CZA), meropenem-vaborbactam (MVB), imipenem-relebactam (I/R) have been introduced onto the market as options to treat infections caused by carbapenem-resistant Enterobacterales. With resistance emerging against these agents, we assessed the *in vitro* activity for cefiderocol (CFDC), a siderophore conjugated cephalosporin, against isolates that were collected between 2020-2022 in Europe and the USA as part of the SENTRY Antimicrobial Surveillance Program that were non-susceptible CZA, MVB, or I/R.

**Table 1.**
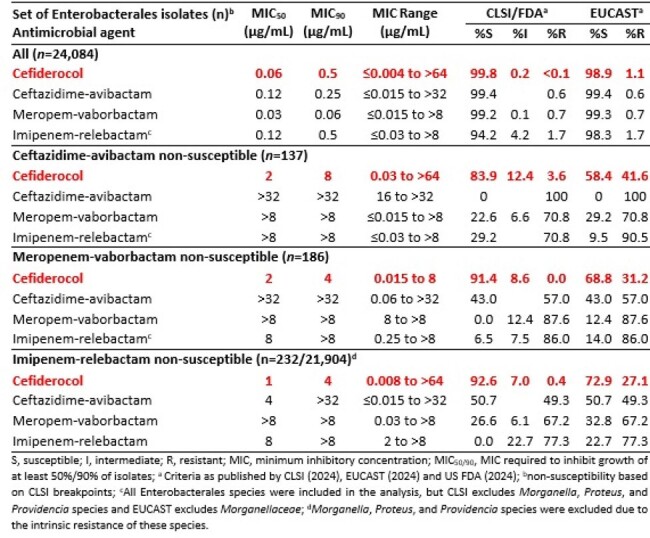
Activity for cefiderocol, ceftazidime-avibactam, meropenem-vaborbactam, and imipenem-relebactam against various non-susceptible phenotypic subsets of Enterobacterales isolates collected from European and US hospitals as part of the SENTRY surveillance program (2020-2022).

**Methods:**

A total of 24,084 Enterobacterales were collected and minimum inhibitory concentrations (MIC) for CZA, MVB, I/R were determined according to CLSI guidelines using broth microdilution with cation-adjusted Mueller-Hinton broth (CAMHB), while for CFDC iron-depleted CAMHB was used. Susceptibility was assessed according to 2024 CLSI, FDA, and EUCAST breakpoints. Non-susceptibility to CZA, MVB, I/R was assessed using CLSI breakpoints.

**Results:**

Few isolates collected were non-susceptible to CZA (0.6%), MVB (0.8%), or I/R (1.0%). 83.9%, 91.4% and 92.6% of CZA-NS, MVB-NS, and I/R-NS isolates tested susceptible for CFDC, according to CLSI breakpoints (Table 1). These percentages were lower when EUCAST breakpoints were applied; 58.4%, 68.8% and 72.9%, respectively, a result of a significant number of isolates with CFDC MIC values of 4 µg/mL. Regardless of the breakpoints used, susceptibility to CFDC was higher compared to any of the β-lactam-β-lactamase inhibitor combinations, which demonstrated a high degree of cross-resistance. This high degree of cross-resistance could be largely explained by the presence of metallo-β-lactamases in CZA-NS (126/137), MVB-NS (104/186) and I/R-NS (113/229) isolates.

**Conclusion:**

CFDC remains active against Enterobacterales that are non-susceptible against β-lactam-β-lactamase inhibitor combinations. In contrast, cross resistance was observed for the β-lactam-β-lactamase inhibitor combinations. CFDC should be considered as a treatment option when Enterobacterales non-susceptible to β-lactam-β-lactamase inhibitor combinations are encountered.

**Disclosures:**

**Boudewijn L. DeJonge, PhD**, Shionogi Inc.: Employee **Sean T. Nguyen, PharmD**, Shionogi Inc.: Employee **Jason J. Bryowsky, PharmD, MS**, Shionogi: Employee **Rodrigo E. Mendes, PhD**, JMI: RM is an employee of JMI. JMI was contracted by and received financial support from GSK to conduct gepotidac|Paratek Pharmaceuticals: Advisor/Consultant|Paratek Pharmaceuticals: Grant/Research Support **Christopher M. Longshaw, PhD**, Shionogi BV: Employee **Miki Takemura, n/a**, Shionogi & Co., Ltd.: Employee **Yoshinori Yamano, PhD**, Shionogi & Co., Ltd.: Employee

